# Supplementary material for: A Structural Equation Model Demonstrating the Relationship between Food Safety Background, Knowledge, Attitudes and Behaviour among Swedish Students
Source: Foods. 2022 May 28;11(11):1595. doi: 10.3390/foods11111595 (PMC9180796; doi:10.3390/foods11111595)
Supplement: Supplementary file 1 [file foods-11-01595-s001.zip › foods-1715677-supplementary.pdf]

Supplemental Table S1: Respondents attitudes towards food safety behavior

**A1**	**Question: To wash your hands carefully before cooking food is for you**? Responders (*n* = 606): Not at all important (0.3%); Not especially important (3.6%); Either important nor unimportant (4.6%) Rather important (29.7%); **Very important (61.7%)** I have never been in this situation (0%).
**A2**	**Question: To wash your hands carefully after handling raw, minced meat is for you?** Responders (*n* = 606): Not at all important (0.5%); Not especially important (1.2%); Either important nor unimportant (3.5%) Rather important (18.8%); **Very important (64.9%)** I have never been in this situation (11.2%).
**A3**	**Question: To wash your hands carefully after handling raw chicken is for you?** Responders (*n* = 606): Not at all important (0.3%); Not especially important (0.2%); Either important nor unimportant (0.5%) Rather important (5.1.%); **Very important (82.3%)** I have never been in this situation (11.6%).
**A4**	**Question: To wash your hands carefully after visiting the toilet is for you?** Responders (*n* = 606): Not at all important (0.0%); Not especially important (0.0%); Either important nor unimportant (0.8%) Rather important (8.9%); **Very important (90.3%)** I have never been in this situation (0%).
**A5**	**Question: To wash your hands carefully after handling raw eggs is for you?** Responders (*n* = 606): Not at all important (4.8%); Not especially important (13.7%); Either important nor unimportant (24.6%) **Rather important (27.4.%)**; Very important (22.4%) I have never been in this situation (4.6%).
**A6**	**Question: To cool leftovers within 4 hours from cooking is for you?** Responders (*n* = 606): Not at all important (3.6%); Not especially important (7.6%); Either important nor unimportant (30.7%) **Rather important (31.2.%);** Very important (26.7%) I have never been in this situation (0.2%).
**A7**	**Question How do you evaluate your level of food safety knowledge?** Responders (*n* = 606): Very good (9.1%); **Rather good (52.6%);** Neither good nor bad (27.6%); Rather bad (8.4%); Very bad (1.7%); Do not know (0.7%).
